# *Thrichomys laurentius* (Rodentia; Echimyidae) as a Putative Reservoir of *Leishmania infantum* and *L. braziliensis*: Patterns of Experimental Infection

**DOI:** 10.1371/journal.pntd.0000589

**Published:** 2010-02-02

**Authors:** André Luiz Rodrigues Roque, Elisa Cupolillo, Renato Sergio Marchevsky, Ana Maria Jansen

**Affiliations:** 1 Laboratory of Tripanosomatid Biology, Oswaldo Cruz Institute, Fiocruz, Rio de Janeiro, Brazil; 2 Laboratory of Leishmaniasis Research, Oswaldo Cruz Institute, Fiocruz, Rio de Janeiro, Brazil; 3 Laboratory of Neurovirulence, Institute of Technology on Immunobiological/Biomanguinhos, Fiocruz, Rio de Janeiro, Brazil; Institut Pasteur de Tunis, Tunisia

## Abstract

The importance of the genus *Thrichomys* in the retention of infection and transmission of *Leishmania* species is supported by previous studies that describe an ancient interaction between caviomorphs and trypanosomatids and report the natural infection of *Thrichomys* spp. Moreover, these rodents are widely dispersed in Brazil and recognized as important hosts of other tripanosomatids. Our main purpose was to evaluate the putative role of *Thrichomys laurentius* in the retention of infection and amplification of the transmission cycle of *Leishmania infantum* and *L. braziliensis*. Male and female *T. laurentius* (n = 24) born in captivity were evaluated for the retention of infection with these *Leishmania* species and followed up by parasitological, serological, hematological, biochemical, histological, and molecular assays for 3, 6, 9, or 12 months post infection (mpi). *T. laurentius* showed its competence as maintenance host for the two inoculated *Leishmania* species. Four aspects should be highlighted: (i) re-isolation of parasites 12 mpi; (ii) the low parasitic burden displayed by *T. laurentius* tissues; (iii) the early onset and maintenance of humoral response, and (iv) the similar pattern of infection by the two *Leishmania* species. Both *Leishmania* species demonstrated the ability to invade and maintain itself in viscera and skin of *T. laurentius*, and no rodent displayed any lesion, histological changes, or clinical evidence of infection. We also wish to point out the irrelevance of the adjective dermotropic or viscerotropic to qualify *L. braziliensis* and *L. infantum*, respectively, when these species are hosted by nonhuman hosts. Our data suggest that *T. laurentius* may act at least as a maintenance host of both tested *Leishmania* species since it maintained long-lasting infections. Moreover, it cannot be discarded that *Leishmania* spp. infection in free-ranging *T. laurentius* could result in higher parasite burden due the more stressing conditions in the wild. Therefore the tissular parasitism of the skin, infectiveness to the vector, and amplification of the transmission cycle of both *Leishmania* species could be expected.

## Author Summary

For *Leishmania*, one genus among several genera belonging to the parasitic Trypanosomatidae family, many nonhuman mammals are known to be hosts in addition to humans. Most studies that describe *Leishmania* wild reservoirs are based on isolated descriptions of infection that can lead to misinterpretation of information. The definition of the epidemiological importance of a putative reservoir host depends on adequate data on the dynamics and peculiarities inherent to the host-parasite interactions and their involvement in the transmission cycle of these parasites. Our objectives were to sort out the features displayed by nonhuman mammal populations (the caviomorph rodent *Thrichomys laurentius*) which, with an insect host, perpetuate *Leishmania* transmission cycles. This rodent species had the ability to act as maintenance and/or amplifier host of both tested *Leishmania* species. The similar pattern of infection displayed by *T. laurentius* infected by these two *Leishmania* species shows that the definition of dermotropic or viscerotropic based on the clinical features observed in humans should not be applied to natural hosts, and it emphasizes that the search for *Leishmania* reservoirs should consider all possibilities of the infection course, independent of current knowledge in other mammal hosts.

## Introduction

Although recognized as one of the most important and widespread parasitic diseases in the world, leishmaniasis prevention and control remains a challenge for health authorities in some countries [1]. In Brazil, human cutaneous leishmaniasis occurs in association with different *Leishmania* species, but *Leishmania (Viannia) braziliensis* is the most frequent and widespread species in the country. The visceral form is exclusively associated with *Leishmania (Leishmania) infantum* (syn. *L.* (*L.*) *chagasi*).

The *Leishmania* genus comprises more than 20 vector-borne species, primarily enzootic parasites, which includes species capable to infect a broad range of mammalian hosts and to be transmitted a variety of phlebotomine vectors. The transmission cycles of *Leishmania* spp. involves a variety of phlebotomine vectors and mammalian hosts. Failure to interrupt human transmission and prevent new epidemics are related, among others, to the involvement of wild and synanthropic hosts, mainly rodents and marsupials, that can colonize peri-urban areas [2]–[4].

Till now, the majority of studies that point out *Leishmania* spp. wild reservoirs are based on punctual observations of infection, most of them by molecular methods (PCR) rather than by parasite isolation and characterization. This can conduct to misinterpretation of concepts since the mere finding of *Leishmania* DNA in a given mammal species is not sufficient to consider this species a reservoir host [5],[6]. Reservoir is better defined not as a single species, but as an assemblage of species responsible for the long lasting maintenance of a parasite in a given environment [7],[8]. This concept does not include target species (human or domestic mammals) neither does it consider the eventual symptoms displayed by the reservoir hosts. Natural *Leishmania* sp. infection in wild rodents was already reported in different parts of the world [2],[9],[10], and some of them were also successful in demonstrating the persistence of infection up to two years [11]–[13]. Laboratory studies using natural hosts as experimental models provide a suitable indication of the importance of these hosts as reservoirs, since it allows a better understanding of the dynamics of infection, especially concerning the ability to retain the infection and amplify the parasite populations in a given environment, due to a feature that favors the parasite transmission (e.g., presence of parasites in the skin). There are only rare studies that follow up experimentally infected wild hosts by *Leishmania* species, mostly due to the difficulties of managing wild mammals in captivity.

*Thrichomys laurentius* is a South American caviomorph rodent formerly included in a monospecific genus. The formerly recognized species, *Thrichomys apereoides*, was recently split into five species: *T. apereoides*, *T. laurentius*, *T. pachyurus*, *T. inermis* and *T. sp* [14],[15]. The recently described species within this genus comprise crepuscular and scansorial rodents that inhabit open vegetation in various Brazilian biomes: savannah (“Cerrado”), white shrub (“Caatinga”) and marshland (“Pantanal”), widespread from western to northern Brazil [16].

Some reasons point to the importance of the *Thrichomys* genus as a putative reservoir for *Leishmania* species: 1) the probable ancient association between caviomorph rodents and the trypanosomatids. It was proposed that the entry of new species of *Leishmania (Leishmania)* subgenus was the consequence of the arrival of infected caviomorph rodents during the Oligocene [17]; 2) the detection of *Leishmania* sp. DNA in free ranging *Thrichomys* sp. These rodents were found infected by *Leishmania* species from different complexes – *L. mexicana* and *L. donovani* – in an endemic area for both visceral and tegumentar leishmaniasis in Minas Gerais state, Brazil [3]; 3) the importance of these rodents as reservoirs of other trypanosomatids – *Trypanosoma cruzi* and *T. evansi*. This feature is confirmed by both experimental [18],[19] and field work studies [20],[21]; and 4) *Thrichomys* spp are widely dispersed throughout Brazil, comprising one of the most abundant species in the three Brazilian biomes where they occur [16]. Moreover, they are habitat generalists, found even in degraded areas, and can also frequent human dwellings [20],[22].

In the present work, we investigated the experimental infection of *Thrichomys laurentius* with *Leishmania infantum* and *L. braziliensis*. Our main purpose was to evaluate the putative role of *T. laurentius* for the retention of infection and amplification of the transmission cycle of these *Leishmania* species. To achieve this aim, we: (i) studied the differences on the course of infection on *L. infantum* and *L. braziliensis* experimentally infected *T. laurentius*; (ii) followed up the health status of experimentally infected rodents by hematological and biochemical parameters, in order to evaluate the consequence on rodents' health of the experimental infection; and (iii) analyzed the parasitism distribution in the host.

## Materials and Methods

### Experimental infection

Twenty-four *Thrichomys laurentius* of both sexes born in captivity were kindly supplied by Dr. Paulo D'Andrea. The colony of *T. laurentius* was derived from 9 males and 38 females captured in Piauí state (northeast region of Brazil) in 2000. The animals are free from other parasites, provided from food and water *ad libitum* and kept under conventional conditions (temperature 24±2°C, natural daylight) at animal facilities of the Laboratory of Biology and Parasitology of Small Reservoir Mammals, Oswaldo Cruz Institute. Animals were individually housed in 41-34-17 cm polycarbonate cages with sawdust as bedding and fed with NUVILAB CR1 mouse pellets (Nuvital nutrients S.A., Colombo/PR, Brazil) [23].

The rodents were divided into two groups and intradermically inoculated into the right ear pinna (0.05mL maximum volume) by either *Leishmania infantum* – MHOM/BR/2001/HP-EMO = IOC-L2504 (n = 12) or *L. braziliensis* – MHOM/BR/2000/LTCP13396 = IOC-L2483 (n = 12) obtained from the Oswaldo Cruz Institute *Leishmania* collection (Coleção de *Leishmania* do Instituto Oswaldo Cruz, CLIOC). At 60 day-old, animals were infected with 10^6^ promastigotes derived from stationary phase culture starting from freshly amastigotes and followed up for 3, 6, 9 or 12 months post infection (mpi). The age of the animals at the time of inoculum was based on calculations from the weightless *T. laurentius* caught in nature, *i.e.*, when young rodents starts to be exposed to infections outside their nest (personnal observations). The parasites (isolated no more than 2 weeks before *Thrichomys* infection) were maintained by *in vivo* passage in golden hamsters (*Mesocricetus auratus*) derived from the animal facilities of Oswaldo Cruz Foundation. In this case, promastigotes were intradermically inoculated in hamsters footpads and re-isolated from inoculation site (*L. braziliensis*) and spleen *(L. infantum)* 4–5 months after infection. Hamsters were also used for control of the infectivity of the inocula.

The study design was carried out according to the protocol approved by the Institutional Committee for Experimentation and Care of Research Animals (CEUA-Fiocruz: P0076/01 and P0269/05) and animal facilities are supported by the Brazilian Institute of Environment and Renewable Natural Resources (IBAMA license 02022.002062/01-04). The present study was conducted from November 2005 to December 2008.

### Follow up of experimental infection

Blood samples were collected in heparinized and nonheparinized tubes from the retro-orbital plexus of animals previously intramuscularly (IM) anesthetized with 100 mg/kg ketamine hydrochloride and 2 drops of local anesthesia with colirium (0.5% solution of proximetacaine chloridrate) every 3 weeks. The following parameters were determined: (i) red (RBC) and white (WBC) blood cell count, using a Neubauer hemocytometer; (ii) hematocrit, by the centrifugation of microcapilar tubes; (iii) hemoglobin levels, using a commercial test kit (Labtest, Lagoa Santa/MG, Brazil); (iv) percentage of leukocyte cells, by microscopical observation of thin blood smears stained with Panótipo Rápido (derived from the Romanowski stain). Heparinized blood was also collected onto filter paper (Whatman 5, Maidstone, UK) for the molecular assay, while the serum obtained from non-heparinized blood samples was used for the biochemical and serological follow-up. Medium corpuscular volume (MCV), medium corpuscular hemoglobin (MCH) and medium corpuscular hemoglobin volume (MCHV) were also calculated. Values of all parameters obtained for each group one-day before the inoculum were considered normal and used to calculate the reference values.

The ability to produce nitric oxide was evaluated by the nitrite level in rodent sera, using the Griess Reagent System (Promega, Madison, USA). Only for rodents infected by *L. infantum*, albumin and total protein levels were determined using commercial test kits (Labtest, Lagoa Santa/MG, Brazil). All of these assays were done according to the manufacturers recommendations.

The kinetics of the humoral immune response was evaluated by indirect immunofluorescence test (IFAT) and enzyme-linked immunosorbent assay (ELISA) using *Leishmania* antigen deriving from axenic promastigotes of the same strain used for the experimental infection (homologous) and/or deriving from a mixture of *L. infantum* and *L. braziliensis* formalin-treated promastigotes (mix), the latter only for the rodents followed for more than 6 months. IFAT was performed assaying two-fold sera dilutions (1∶10–1∶1,280) against *Leishmania* promastigotes and the reactions conducted using a specific in-house intermediary antibody anti-*Thrichomys* sera produced in rabbits. The reaction was visualized using a commercial anti-rabbit IgG-FITC (Sigma-Aldrich, St. Louis, USA), according to Camargo [24]. Standard micro-ELISA was conducted according to Rosario *et al.* [25], using a commercial anti-rat IgG-peroxidase (Sigma-Aldrich, St. Louis, USA). We established 1∶20 and 1∶30,000 for the sera and conjugate dilutions, respectively, after the analysis of different serum dilutions derived from experimentally infected and non infected captivity *Thrichomys* using the ROC Curve (BioStat 5.0 software). For each rodent, the cut-off value was determined using sera collected before the experimental infections and the absorbance at 492 nm was measured in an EMax Microplate Reader (Molecular Devices, Ramsey, USA).

The DNA extraction from filter paper was conducted by boiled water, according to Marques *et al*. [26]. The DNA product amplifications were conducted using pureTaq Ready-To-Go PCR beads (Amersham Biosciences, Buckinghamshire, UK) and primers directed to the conserved region of the *Leishmania* kDNA minicircle, as follows: forward: 5′-GGGGAGGGGCGTTCTGCGAA-3′ and reverse: 5′-GGCCCACTAT ATTACACCAACCCC-3′. The PCR conditions were as follows: initial denaturation at 94°C for 5 min, followed by 30 cycles at 94°C for 1 min, 60°C for 1 min, 72°C for 30 s, and a final extension at 72°C for 5 min [27]. Blood from uninfected rodents and uninfected blood samples to which promastigotes axenically cultured were added, were used as control for both extraction and amplification processes. The amplified polymerase chain reaction (PCR) products were analyzed in polyacrylamide gel electrophoresis (4%) and the negative samples were re-analyzed by electrophoresis in 12.5% polyacrylamide gels using the Genephor electrophoresis system apparatus (Pharmacia Biotech). All of the gels were stained using the DNA Silver Staining Kit (GEHealthcare, Chalfont St. Giles, UK).

### Necropsy procedures

Euthanasia was performed by CO_2_ inhalation on months 3, 6, 9 and 12 post inoculation (n = 3 for each batch). Procedures were undertaken in a Class II biosafety cabinet: (i) inoculation of fragments of spleen, liver, inoculation site (right pinna skin) and bone marrow in biphasic culture mediums (NNN/Schneider's) supplemented with 10% fetal bovine serum (v/v) and antibiotics (350 IU penicillin and 150 µg/mL streptomycin), which was examined every 3–4 days for 1 month; (ii) slide imprints of spleen, liver and inoculation site, which were Giemsa-stained and microscopically observed at ×400 magnification; (iii) collection of tissue fragments – spleen, liver, inoculation site, skin and bone marrow – in 1.5 mL tubes containing ethanol and stored at −20°C, which were used for the molecular analyses; (iv) fixing of tissue fragments – spleen, liver, skin, lymph nodes and both ears separately – in 10% neutral buffered formalin for histological studies. After dehydration and paraffin-embedding, 4 µm sections in thickness were made, routinely stained with hematoxylin and eosin (H&E), and the sections examined by light microscopy. For the slide imprints, histological and molecular tests, liver tissue samples were performed considering two fragments from different lobes.

For the molecular diagnosis, tissue fragments were washed three times with Milli-Q water and DNA extraction realized using the Wizard Genomic DNA Purification Kit (Promega, Madison, USA) according to the manufacturer's recommendations. The PCR was conducted as described above for the blood collected on filter paper.

### Data analysis

Normal ranges for the hematological and biochemical values were determined in relation to medium values and two-fold standard errors obtained for each group one-day before the inoculum. The differences on the hematological and biochemical kinetics between rodents infected by either *L. braziliensis* or *L. infantum* were evaluated by the non-parametric Mann-Whitney test. The differences between the normal values and each point of the hematological and biochemical follow-up were evaluated by the Kruskal-Wallis and Student-Newman-Keuls tests. All of the data were analyzed using the BioStat 5.0 software (Instituto Mamirauá, Tefé, Brazil) and considering p<0.05 significant.

## Results

*Thrichomys laurentius* was able to control and retain the infection for both inoculated *Leishmania* species (*L. braziliensis* and *L. infantum*), and four aspects should be highlighted: (i) the long-term retention of *T. laurentius* infection, at least 12 months; (ii) the low parasitic burden displayed by *T. laurentius* tissues on the necropsy; (iii) the early onset and maintenance of important humoral response, demonstrated by significant serological titers; and (iv) the similar pattern of infection displayed by *T. laurentius* infected by these two *Leishmania* species usually associated to distinguishable manifestations of human disease. This latter aspect was mainly demonstrated by the parasite recovery from internal viscera and detection of *Leishmania* DNA in all sampled tissues of *L. braziliensis* infected rodents. This is the first report of an experimental infection in a putative wild rodent reservoir species, where *L. braziliensis* and/or *L. infantum* could be re-isolated.

### Kinetics of infection

The *Leishmania* sp. inoculated were shown to be infective as demonstrated by the parasite recovery from liver, spleen and inoculation site of hamsters. However, only *L. braziliensis* could be isolated from the inoculation site. As expected for outbred animals, a great individual variability among infected *T. laurentius* was noted. Despite that, all *Thrichomys* rodents were able to efficiently control the infection without presenting lesion or clinical evidence of disease. Growth development, determined by weekly body mass measure, was not affected by the *Leishmania* infection and no expressive alterations of health markers were observed. Amastigotes of *Leishmania* spp. were absent in the thin blood smears and *Leishmania* DNA could not be detect in blood samples collected in filter paper in 3 weeks interval.

**Figure 1:**
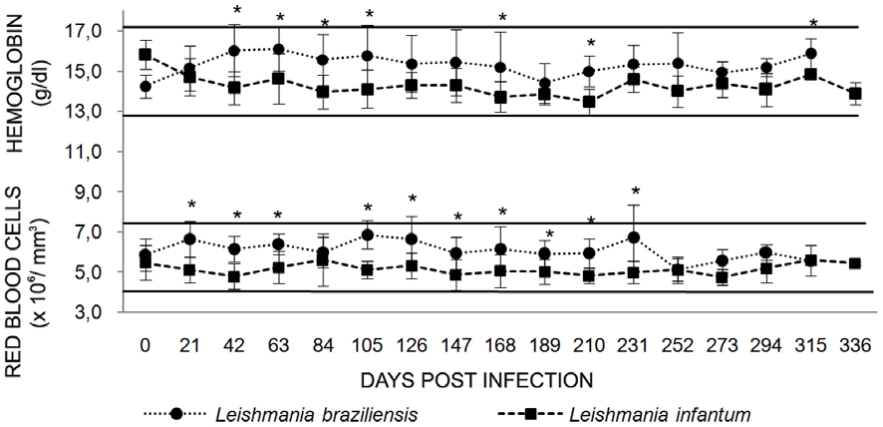
Kinetics of red blood cell counts and hemoglobin levels in *Thrichomys laurentius* experimentally infected by *Leishmania braziliensis* (Lb) or *Leishmania infantum* (Li). Each point represents the mean value and standard errors. The continuous lines indicate the normal range defined by the medium values obtained for each group one-day before the inoculum and two-fold standard errors. The * indicates the significant difference between rodents infected either by *L. braziliensis* or *L. infantum*.

**Figure 2:**
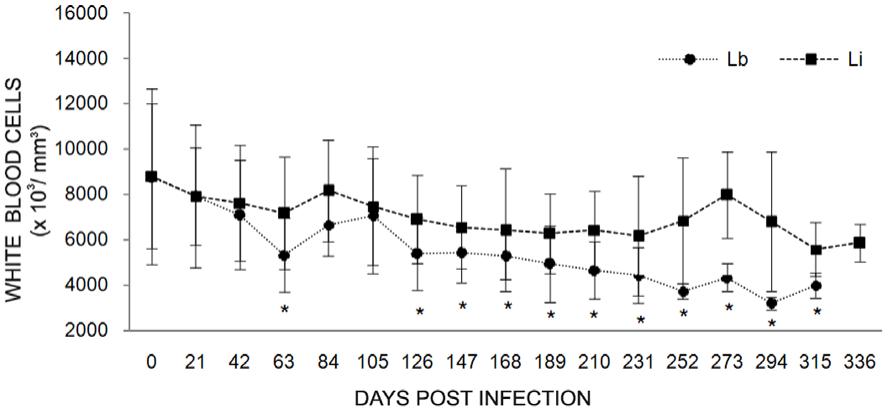
Kinetics of white blood cell counts in *Thrichomys laurentius* experimentally infected by *Leishmania braziliensis* (Lb) or *Leishmania infantum* (Li). Each point represents the mean values and standard errors. The * indicates the significant difference between values obtained from non-infected rodents (day 0) and and rodents infected by *L. braziliensis*.

*Leishmania* infection did not result in anemia and all of the rodents displayed values that were inside the normal range during the complete follow-up. Nevertheless, *L. infantum* infected *T. laurentius* tended to display lower red blood cell counts and hemoglobin levels when compared to those infected by *L. braziliensis*. During the follow-up, most of the values obtained for RBC counts and hemoglobin levels showed significant differences (p<0.05) between *T. laurentius* infected by *L. braziliensis* and *L. infantum* (Figure 1). This same feature was also observed for the hematocrit values (data not shown). Significant leucopenia from the 120 dpi day on (p<0.05), was observed in rodents inoculated with *L. braziliensis*. A similar, but not significant, picture was also observed in the rodents infected by *L. infantum* (Figure 2). No differences before and after the inocula were observed for MCV, MCH, MCHV and differential counts of WBC (data not shown).

**Figure 3:**
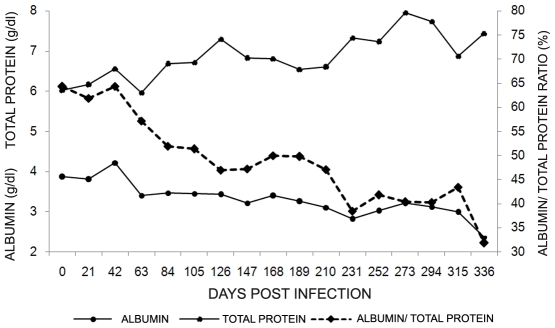
Kinetics of albumin and total protein levels and albumin/total protein ratio in a *Leishmania infantum* infected *Thrichomys laurentius* (7548). Day 0 indicate the values obtained before the experimental infection.

Albumin and total protein levels were not affected by *L. infantum* infection, excepting for the rodent (7548) where re-isolation of parasites was possible. This rodent displayed a marked decrease in albumin levels and increase in total protein levels after 200 dpi, resulting in declined in albumin/total protein level (Figure 3). The nitrite level in rodent sera displayed a great individual variability and could not be correlated to any other hematological, serological or parasitological parameter.

**Figure 4:**
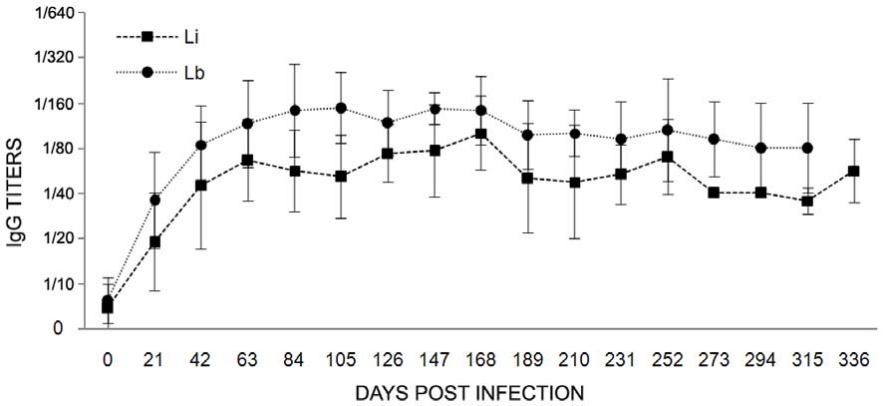
Kinetics of the humoral immune response (IFAT) in *Thrichomys laurentius* experimentally infected by *Leishmania braziliensis* (Lb) or *Leishmania infantum* (Li). Each point represents the mean value and standard errors of the IgG titers of infected rodents.

All infected *T. laurentius* were able to produce a humoral response that could be detected during the three initial weeks by both IFAT and ELISA assays. The IFAT showed no differences on serological titers among assays performed with homologous or a mixture of antigens. The response onset and magnitude of titers were very homogeneous and similar among the infected rodents. Rodents infected by *L. braziliensis* displayed serological titers that were always slight higher than those observed for the rodents infected by *L. infantum* (Figure 4). The ELISA assay revealed an individual variability on the response onset and magnitude of titers that varied from negligible to strong responses. In common, infected *T. laurentius* displayed a peak of absorbance values on 100 dpi that were in medium four times higher than the day 0 and kept constant until the end of follow-up (data not shown).

### Necropsy

Both *Leishmania* species demonstrated the ability to invade and maintain itself on viscera and skin of the infected *T. laurentius*, although this parasitism was not expressive since isolation of parasites was rare: from liver, spleen and inoculation site 3 mpi from one rodent infected by *L. braziliensis*; and from liver and spleen 12 mpi from one rodent infected by *L. infantum*.

*Leishmania* DNA was detected in all experimental batches, independent of the *Leishmania* species (Table 1). Nevertheless, the low parasitic burden was evidenced by the large amount of positive PCR (73%) observed only when electrophoresis was conducted on the GenePhor electrophoresis system apparatus. Up to 77% and 50% of the *L. braziliensis* and *L. infantum* infected *T. laurentius*, respectively, displayed *Leishmania* DNA in at least one of the tissues collected on the necropsy. The individual variability, peculiarities of the host, parasite and host-parasite interaction, and the time of the infection seems to be the major factors that influenced the different percentage of positive reactions. Parasite distribution in viscera was not homogeneous and 30.4% of the tissues (spleen, liver and inoculation site) that had two different fragments examined, displayed a positive and a negative result for the presence of *Leishmania* DNA.

**Table 1:** Detection of *Leishmania* sp. DNA by polimerase chain reaction (PCR) in different tissues sampled from *Thrichomys laurentius* experimentally infected by *Leishmania braziliensis* or *Leishmania infantum* between months 3 and 12 post infection.

Infected group	*Leishmania braziliensis*				*Leishmania infantum*			
Tissue/Necropsy date	3mpi	6mpi	9mpi	12mpi	3mpi	6mpi	9mpi	12mpi
Spleen	4/6	1/3	0/3	0/3	0/3	0/2	0/3	1/3
Liver	6/8	4/6	3/6	2/6	2/6	0/4	0/6	1/6
Inoculation site	4/6	1/3	0/3	0/3	0/3	0/2	0/3	1/3
Opposite ear	1/2	0/2	0/3	1/3	0/3	0/2	0/3	0/3
Abdominal skin	5/6	0/3	0/3	0/3	0/3	1/4	0/3	1/3
Bone marrow	1/2	0/3	0/2	0/3	0/3	0/2	1/3	3/3

Each point represents the number of positive/total PCR reactions observed for all tissue samples in the different experimental batches.

mpi. months post infection.

**Figure 5:**
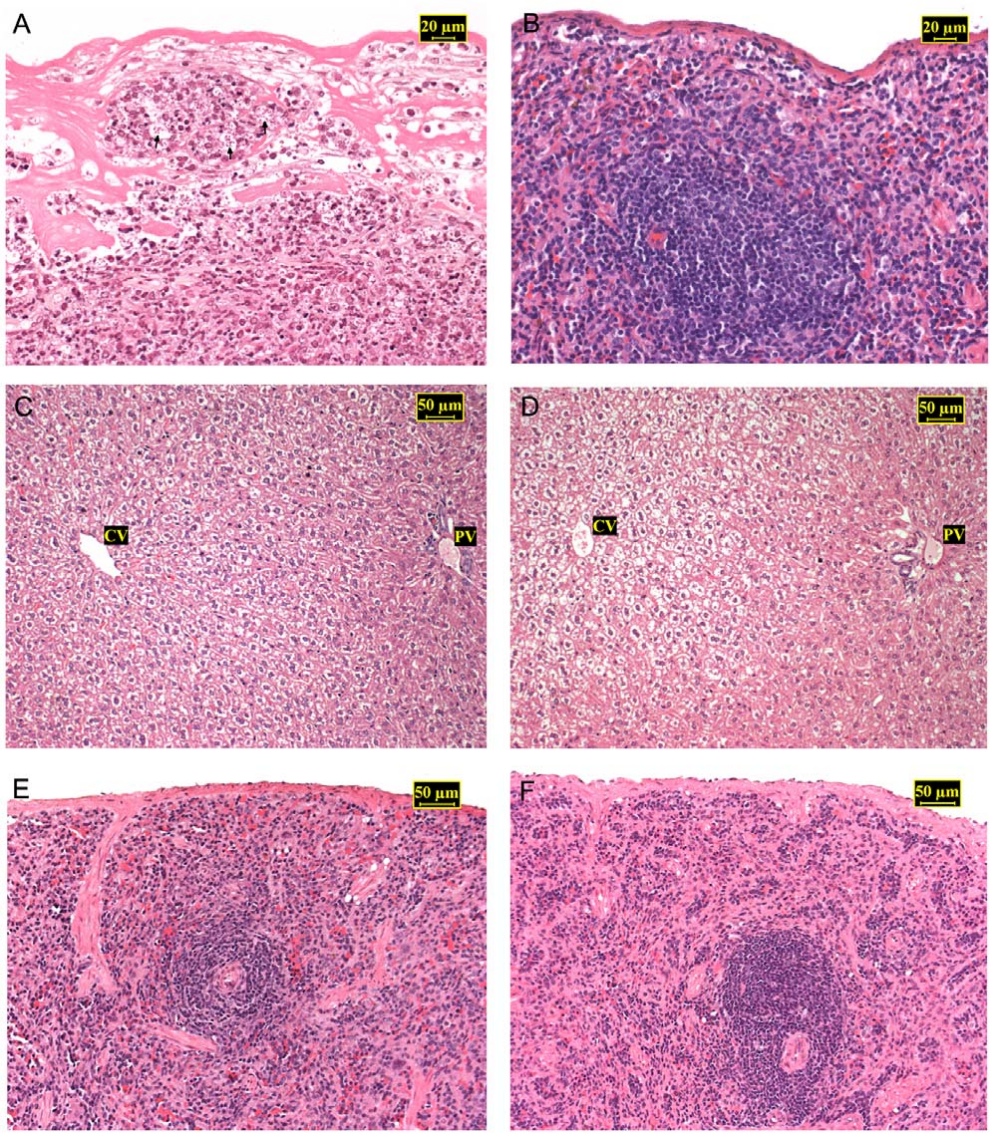
Photomicrographs of spleen and liver sections from *Leishmania braziliensis* or *L. infantum* infected rodents stained with haematoxylin-eosin. A: *L. braziliensis*-infected golden hamster (*Mesocricetus auratus*) 3 months post infection (mpi) – spleen revealed extensive necrosis and inflammation. Note necrotic focus containing amastigotes (arrows). B: *L. braziliensis*-infected *Thrichomys laurentius* (7289) 3 mpi – spleen showing architecture of red and white pulp, lack of parasites; C: *L. infantum*-infected *T. laurentius* (7548) 12 mpi (liver culture tissue positive) – panoramic view of the liver showing typical architecture with portal (PV) and central vein (CV); D: *L. infantum*- infected *T. laurentius* (7538), 12 mpi (liver culture tissue negative) – also illustrated liver showing normal architecture; E: *L. infantum*-infected *T. laurentius* (7548), 12 mpi (liver culture tissue positive) – overview of white and red pulp from spleen; F: *L. infantum*-infected *T. laurentius* (7538), 12 mpi (liver culture tissue negative) – also illustrated spleen overview of red and white pulp.

No sign of parasitism was observed in tissue imprints or histological sections. Comparative histologic analysis did not detect any inflammatory or degenerative changes in *T. laurentius* infected with *L. infantum* or *L. braziliensis*. Our study on the pathology of *Leishmania* sp. in golden hamster (*Mesocricetus auratus*) demonstrated that this rodent developed definite evidence of infection, characterized by extensive spleen necrosis and inflammation associated to high number of amastigotes (Figure 5A). No histological abnormalities or other histological differences were observed between positive and negative culture tissues obtained from infected *T. laurentius* (Figure 5B–F). Discrete differences in the cellularity of primary splenic follicles and periarterial lymphoid sheath seem not related to infection and were seen in seven *T. laurentius* from both groups.

## Discussion

The genus *Thrichomys* comprises recently described cryptic species that are undergoing a process of allopatric and/or parapatric differentiation [14]. Within this widespread rodent genus, *T. laurentius* is distributed in northeast Brazil, from Ceará to Bahia state, a region that reports numerous cases of both visceral and tegumentar human leishmaniasis [28],[29]. For demonstrating the potential to act as a maintenance host, a given mammal species must be able to control and retain the parasite infection. In our experimental conditions this rodent species showed to be able to retain long term infections by the main etiological agents of human leishmaniasis in Brazil, *Leishmania infatum* and *L. braziliensis*. This ability was undoubtedly demonstrated by the parasite re-isolation in liver and spleen of rodents experimentally infected by both *Leishmania* species.

Asymptomatic infection is usually considered as an essential attribute to be considered a reservoir host. This is currently not considered as a rule; in fact, it is the transmission strategy of the parasite that is positively selected in a successful host-parasite system, independent of the damage caused by the parasite or health status displayed by the host. Even ancient host-parasite interaction may not necessarily evolve in the direction of less damage or lower virulence, but instead of that, to the maximum transmissibility of the parasite [30],[31]. According to the concept proposed by McMichael [32] and Roque *et al.* [19], maintenance host is the one who retain the infection (where a given parasite persists) while an amplifier host displays an infection course that favors the transmissibility of the parasite. Taken together our data suggest that *T. laurentius* may act at least as a maintenance host of both tested *Leishmania* species since it maintained long-lasting infections. Moreover, it cannot be discarded that in nature, infected rodents display higher parasite burden and tissular parasitism on skin, acting then as an amplifier hosts of *Leishmania* species.

Anemia, a characteristic trait in *L. infantum* infected humans, dogs and laboratory rodents [33]–[35], was not observed in the *L. infantum* infected *T. laurentius*. Although animals infected by *L. infantum* displayed a significant decrease of the hematological parameters in comparison to those infected with *L. braziliensis*, this decrease still did not characterize anemia. Leucopenia, another common trait observed on *L. infantum*, but not on *L. braziliensis* infections [36], was only observed in the *L. braziliensis* infected *T. laurentius* from the 120 dpi onwards. This finding was not surprising in the light of the parasite disseminated to liver and spleen. Surprising was (i) the absence of leucopenia in *L. infantum* infected *T. laurentius*; and (ii) the later presence of that leucopenia, only after 120 dpi, while the *L. braziliensis* isolation occurred before that.

Hypoalbuminemia and hypergamaglobulinemia, the most common biochemical alterations in *L. infantum* symptomatic infection in humans and dogs [34],[37], were only observed on the rodent from which the re-isolation of parasites was possible, probably due to a higher parasite burden in this animal. Considering that biochemical alterations are not described as being associated to *L. braziliensis* infections, these parameters were not tested in the animals infected by this *Leishmania* species. Moreover, given the similar pattern observed in the rodents infected with both *Leishmania* species we question whether analyses of further parameters could display alterations in the animal from which *L. braziliensis* was isolated in the necropsy.

Experimental *T. laurentius* infection by two different *Leishmania* species did not result in important damage for rodents, but this can be quite different for naturally infected *T. laurentius*. Captivity rodents are free from other pathogens, are provided food and water *ad libitum* and maintained in controlled environmental conditions. In nature, rodents are constantly facing out stress (search for food and escape from predators), competitions (intra and inter-specific), and infection by other parasites and *Leishmania* re-infections. All of these factors will directly influence the course of any parasitic infection and be reflected by higher virulence and/or host damage.

The effectiveness of the serological assays was demonstrated even for the rodents infected by *L. braziliensis*, an infection usually not associated to an important humoral response [36],[38]. This is probably due to the visceralization of this parasite species in *T. laurentius*. This data emphasizes that the search for *Leishmania* reservoir should consider all possibilities of the infection course, which includes a broad range of diagnostic methods independent of the current knowledge in other mammal hosts. The efficacy of the IFAT assay for serological screening of *Thrichomys* sp. was already demonstrated for *Trypanosoma cruzi* and *T. evansi* infections [19],[39]. In the present study, ELISA showed to be a promising tool, since it was able to detect a humoral response production in all of the infected rodents. The use of an intermediate anti-*Thrichomys* antibody and the determination of cut-off values based on a great number of positive and negative serum samples might result in a standardized and efficient assay to diagnose *Leishmania* infection in wild *Thrichomys* sp.

In this study, we were not able to detect *Leishmania* DNA in any of the blood samples examined; even considering that 24 infected *T. laurentius* were analyzed and blood samples were collected every 21 days post infection, totalizing 236 samples evaluated. These data show that whole blood is not a reliable sample to detect *Leishmania* infection, at least in this mammal host species. The persistence of both *Leishmania* species with an extremely low burden in *T. laurentius* could only be demonstrated by the use of a more sensitive technique: PCR targeting a high copy number DNA sequence coupled to a high resolution electrophoresis (in this study, the Genephor electrophoresis system). Unfortunately, the elevated cost of some commercial kits makes the routine use still unfeasible.

In *T. laurentius*, *L. braziliensis* can invade and maintain itself in other tissues in addition to the skin. The parasite's ability to invade and maintain itself in internal organs, such as spleen and liver, in non-human hosts was described several times since the 1950^ths^ [40],[41]. Despite that, the description of *L. braziliensis* as a dermotropic parasite is widespread throughout the scientific community. Our results demonstrated that the definition of dermotropic or viscerotropic based on the clinical feature observed in humans should not be applied to the natural hosts of that *Leishmania* species.

Studies based only on molecular probes are successful to determine parasite hosts, but lack the capacity to determine the transmissibility of that parasite, and thus the importance of that putative reservoir host on the transmission cycle. Moreover, contamination in isolation attempts in field conditions seriously hampers the successful isolations. For that reasons only few studies were capable to isolate *Leishmania* parasites in naturally infected wild rodents [2],[9],[42]. The polymerase chain reaction (PCR) methodology is undoubtedly a great advance for the diagnosis of *Leishmania* infection, but it cannot be associated to parasite transmissibility.

The scarce studies on the *L. infantum* experimental infection of wild rodents report the failure to re-isolate the parasite [43]–[45], and only in one of them, parasite DNA could be detected [45]. We were able to re-isolate *L. braziliensis* and *L. infantum* from experimentally infected *T. laurentius*. Moreover, we detected *L. infantum* DNA in bone marrow samples of another species of *Thrichomys*, *T. pachyurus*, one year after the experimental infection (unpublished data). The ability to maintain and disseminate to different organs (which include bone marrow, spleen, liver and skin) during long term infections by *Leishmania* species and their wide and abundant distribution in Brazilian endemic leishmaniasis areas point to the importance of *Thrichomys* spp. at least as maintenance host for *Leishmania* species. Future studies concerning the natural infection of *Thrichomys* spp. becomes crucial to understand the role of these caviomorph species on the wild transmission cycles of *Leishmania* species.
